# Words, words, words

**DOI:** 10.1186/0778-7367-69-1

**Published:** 2011-10-24

**Authors:** H Van Oyen, O Bruyère

**Affiliations:** 1 Public Health and Surveillance, Scientific Institute of Public Health, Brussels, Belgium; 2 Public Health, Epidemiology and Health Economics, University of Liège, Liège, Belgium

## 

'Words, words, words.' This was Hamlet's reply to Polonius' question, 'What do you read, my lord?' (Shakespeare, 1603) [1]. By repeating the word three times, Hamlet suggests that what he is reading is meaningless. Before launching the journal *Archives of Public Health*, we have asked ourselves: 'Do we not already produce too many words - too many journals?' or in other words 'What is the added value of yet another public health journal?'

Meaninglessness can hardly be the aim of *Archives of Public Health*. This new journal wants to compile public health knowledge for action. Texts in *Archives of Public Health *should contribute to a better understanding of the population's health. We expect authors to take the time to think and to highlight in their manuscript the implications of the findings for public health policy, public health practice and/or public health research. Thus, each manuscript will contribute to public health knowledge, enhance the interactions between research, policy and practice and stimulate public health monitoring and the development of indicators.

*Archives of Public Health *is not a totally new journal. The journal was initiated by the Belgian Ministry of Health in the years before the Second World War as the *Belgian Archives of Social Medicine, Hygiene, Occupational Health and Forensic Medicine*. Although the name changed and the official Belgian languages were no longer used by the journal, the journal continued to be supported by the Federal Ministry of Health and became, and still is, the official journal of the Belgian Association of Public Health.

The choice for an open access publishing model was not evident as the model is still controversial. We are however convinced that the advantages surpass the disadvantages. One disadvantage of the open access model is related to the misunderstanding about the Article Processing Charge (APC). Some will argue that a model of pay-to-publish allows authors of bad research to buy their way in. However, APCs only apply to papers surviving the scrutiny of the review process. Another argument often raised against APCs is that a pay-to-publish model obstructs free and open exchange of scientific results. APC could limit the publishing capacity for authors, especially those from developing countries [2]. Therefore, Biomed Central provides automatic waivers for authors of countries classified by the World Bank as low-income or lower-middle-income countries [3]. In this way the open access model is more equitable than any other publishing model as it provides resource-poor countries with both the possibility to publish their research results and the possibility to access existing research, although the access to internet may remain a limiting factor [4]. Research is not finished until it is published and therefore the APC is considered as part of a research budget. Moreover, policies of several funding and government agencies [5].stimulate the open and unrestricted access to published research [6-8].

The free and universal online accessibility ensures that the author's work reaches a much larger set of readers, resulting in a greater number of citations and a higher impact. Open access is likely to accelerate dissemination and uptake of research findings. It has been reported that the odds of being cited for open access articles in comparison with non-open access articles are respectively two times and three times higher in the first 4 to 10 months and 10 to 16 months after publication [9].

*Archives of Public Health *chooses an open peer-review policy. This means that the reviewers know the name of the authors and vice versa and the reviewers' non-confidential comments are published in the pre-publication history, as well as all versions of the manuscript. Using the online submission system, the large public health expertise of the members of the Editorial Board and the manuscript handling system provided by BioMed Central, we hope to keep the time between submission, the review process and editorial decision on a manuscript close to 2 months.

One of the many benefits of moving to BioMed Central is that many of its journals are included in a wide range of indexing services [10]. We hope *Archives of Public Health *will follow suit and be indexed in as many of these as possible.

Public health is a very large domain covering all aspects involved in promoting and achieving healthy populations through informed choices of societies and individuals. Public health is global. We tried to combine these two elements when composing the Editorial Board [11]. The members of the Editorial Board do not only live and work in different continents, their expertise is also a reflection of the various disciplines involved in public health. *Archives of Public Health *tends and wants to be an important information source on applied research in all aspects of public health and welcomes articles on health services research, health economics, community interventions, epidemiological studies dealing with international comparisons, determinants of inequality in health and the environmental, behavioural, social, demographic and occupational correlates of health and diseases.

*Archives of Public Health *is not only interested in the described results but also invites authors to reflect on the meaning and impact of their words: what are the implications of their research article for public health actions, interventions, policies and research?

To understand population health, trends in population health and differences in health between subpopulations, the journal will pay special attention to the methods used to monitor population health and more particularly two special topics that are rarely covered by research journals - health indicator development and public health reporting. Both are essential to go beyond Hamlet's 'words, words, words', and compile public health knowledge for action, the motto of *Archives of Public Health*.
